# Landmark Recognition Beyond Curated Benchmarks: Cross-Domain Evaluation of a Multi-Threshold Selective YOLO11 Ensemble on User-Generated Imagery, with a Zero-Shot Multimodal LLM Baseline

**DOI:** 10.3390/jimaging12080397

**Published:** 2026-08-21

**Authors:** Ulugbek Hudayberdiev, Abdimumin Alikulov, Adkham Israilov, Muhiddin Xidirov, Javokhir Musaev

**Affiliations:** 1 Faculty of Economics, Samarkand State University of Veterinary Medicine, Livestock and Biotechnologies, Samarkand 140103, Uzbekistan; 2 Pre-school and Primary Education Faculty, National Pedagogical University of Uzbekistan Named After Nizami, Tashkent 100185, Uzbekistan; 3 Department of Computer Engineering, Yeungnam University, Gyeongsan 38541, Republic of Korea

**Keywords:** landmark recognition, domain shift, user-generated content, ensemble learning, image enhancement, YOLO11, multimodal large language models, zero-shot evaluation, smart tourism, cultural heritage

## Abstract

Landmark recognition for smart tourism is usually validated on curated benchmark images. In deployment, however, the classifier must handle user-generated photographs whose viewpoint, lighting, resolution, occlusion, and compression differ sharply from curated data. This paper evaluates a previously published multi-threshold enhancement and selective YOLO11n-cls ensemble under this shift, and provides a preliminary zero-shot comparison of three general-purpose multimodal large language models (MLLMs) on the same task. To measure the shift, we build Samarkand v2-SNS, a 300-image out-of-distribution test set of social-media photographs of 12 Samarkand landmarks, disjoint from the training and validation data. Under the shift, four supervised baselines fall by 12.73–22.08 percentage points to 73–80% accuracy, and their in-distribution ranking does not hold. The selective ensemble degrades least (99.24% to 93.00%, −6.24 points) and outperforms the strongest baseline by 13 points. A capacity-matched ablation shows that most of this robustness comes from enhancement diversity, not from generic ensembling. In a preliminary comparison, zero-shot MLLMs (GPT-5, Claude Sonnet 4.5, Gemini 2.5) reach only 24.81–54.26%, far below deployment needs. The results argue for reporting out-of-distribution accuracy alongside curated benchmarks, and for hybrid systems that pair compact specialised recognisers with MLLM-based interpretation.

## 1. Introduction

Automatic recognition of historical landmarks from tourist photographs is now a core sensing capability of smart tourism systems. It links spontaneous photo-taking to real-time information delivery, augmented-reality guidance, visitor-flow analytics, and digital heritage documentation [1,2,3,4]. Because these services run on a smartphone at the moment a photograph is taken, the classifier must meet three requirements at once: high accuracy on fine-grained, visually similar architecture; robustness to uncontrolled acquisition conditions; and efficiency compatible with on-device inference [5,6,7].

In earlier work, we built a compact recognition pipeline for the first and third requirements, targeting a culturally important but under-represented region: the UNESCO World Heritage city of Samarkand, Uzbekistan. We first combined a pixel-intensity enhancement that squares values above a threshold with a two-member YOLO11n-cls ensemble [8]. We then generalised this to a multi-threshold formulation (*k* ∈ {100, 150, 225}) with a selective ensemble that searches all fifteen non-empty subsets of four specialised models and fuses the chosen members by logit averaging [9]. On the curated Samarkand v2 test set, the selective ensemble reaches 99.24% accuracy, exceeding MobileNetV3, ResNet50, EfficientNetB0, and a standalone YOLO11n-cls by 0.49–10.88 points, with the gains of the lower-threshold variants confirmed by paired significance tests [9].

Two questions, though, decide whether such a system is useful in the field, and both were left open.

The first is domain shift. A large body of evidence shows that a model evaluated only on the distribution it was curated from overstates its deployment performance: reconstructed test sets [10], synthetic corruptions [11], in-the-wild shifts [12], dataset bias [13], and shortcut learning [14] all produce accuracy drops that in-distribution metrics do not predict. Smart tourism is a textbook case. The images a deployed system actually receives are user-generated content (UGC) from social and messaging platforms [15]—photographs with arbitrary framing, extreme lighting, crowds, filters, and heavy recompression. Whether the near-perfect curated accuracy of a specialised landmark classifier survives contact with UGC has, to our knowledge, not been measured for Central Asian heritage imagery.

The second is the role of general-purpose multimodal large language models (MLLMs). Vision–language pretraining transfers well to many recognition tasks with no task-specific training [16], and recent frontier systems show broad visual competence [17,18,19]. If an off-the-shelf MLLM could identify regional landmarks reliably from a single prompt, the case for building and maintaining specialised recognisers would weaken. If instead MLLMs fail on fine-grained regional heritage—categories that are thin in web-scale pretraining—then specialised pipelines remain necessary, and MLLMs are better placed as a downstream interpretation layer.

This paper answers both questions with one controlled study: 12-class landmark classification in Samarkand. This is an evaluation study by design: benchmark studies that introduce no new model—the ImageNet reproducibility study [10], the common-corruptions benchmark [11], and WILDS [12]—are an established and influential line of work, precisely because in-distribution accuracy is a poor predictor of field performance. This paper does not introduce a new model. It evaluates the recognition pipeline of [8,9] under two conditions that earlier method-focused studies—including our own—did not test: real user-generated photographs and off-the-shelf multimodal LLMs. The contribution is direct evidence on how the method behaves in deployment. Specifically, we make the following contributions:

**(1) A user-generated out-of-distribution benchmark.** We build Samarkand v2-SNS, a test-only set of social-media photographs of the same 12 landmarks, disjoint from the training and validation data of Samarkand v2, and use it to measure the cross-domain generalisation of five supervised systems trained only on curated imagery.

**(2) A robustness result for enhancement-diverse selective ensembling.** Under the curated-to-UGC shift, the selective ensemble degrades by only 6.24 points, vs. 12.73–22.08 points for four standard architectures, and cuts the UGC error rate of the strongest baseline (EfficientNetB0) by 65% in relative terms (7.00% vs. 20.00% error).

**(3) An ablation that isolates the source of this robustness.** A capacity-matched, seed-diverse ensemble of the same architecture on raw imagery recovers only part of the gain (76.67% to 82.00% on UGC), whereas the enhancement-diverse ensemble reaches 93.00%. Most of the robustness therefore comes from enhancement diversity, not from the generic benefit of deep ensembling.

**(4) Evidence that in-distribution rankings do not transfer.** The best baseline on curated data (YOLO11n-cls, 98.75%) falls behind EfficientNetB0 on UGC (76.67% vs. 80.00%). Although this reversal lies within overlapping intervals, the loss of a six-point curated advantage under shift shows that selecting an architecture on curated validation alone can mislead deployment.

**(5) A preliminary zero-shot MLLM benchmark for fine-grained regional heritage.** Under a constrained 12-way prompt, GPT-5, Claude Sonnet 4.5, and Gemini 2.5 reach 29.46%, 54.26%, and 24.81% accuracy on the curated test set—roughly 45–74 points below the specialised ensemble, whose reference value is computed on the full 131-image split (Section 3.4).

The remainder of the paper is organised as follows. Section 2 reviews related work. Section 3 summarises the recognition framework and describes the construction of Samarkand v2-SNS and the MLLM protocol. Section 4 reports the cross-domain, ablation, and zero-shot results. Section 5 discusses mechanisms, implications, and limitations, and Section 6 concludes.

## 2. Related Work

### 2.1. Landmark Recognition and Datasets

Landmark recognition has moved from local-feature retrieval to deep classification and retrieval at scale, with attentive local features [20] and the Google Landmarks Dataset v2 [21] setting large, long-tailed benchmarks. At web scale, the dominant paradigms are instance-level retrieval against a labelled gallery [20,22] and photo geo-localisation from consumer photographs [23,24]; both share our deployment condition of uncontrolled user imagery. For a fixed regional inventory of 12 sites, however, closed-set classification allows compact on-device deployment, which is the setting we study. Classification backbones from AlexNet [25] and ResNet [26] to efficiency-oriented designs such as MobileNetV3 [5] and EfficientNet [6] provide standard accuracy–efficiency trade-offs, while one-stage detector families—most recently YOLO11 with its classification variant YOLO11n-cls [27,28,29]—target real-time mobile inference. Deep models have also been applied directly to architectural heritage imagery [30]. The geographic coverage of public landmark datasets is skewed, however: Google Landmarks Dataset v2 inherits the coverage biases of its web-sourced imagery [21], and Central Asian destinations are sparse in mainstream benchmarks, a gap also documented for other non-Western destinations [31]. The Samarkand and Samarkand v2 datasets [8,9] address this regional gap with, respectively, 9 and 12 categories of Timurid-era and later Islamic architecture, whose dense glazed tilework, repeated geometric ornament, and strong illumination contrasts produce high intra-class variability and inter-class similarity.

### 2.2. Robustness Under Distribution Shift

That in-distribution accuracy overstates real-world performance is well documented. Torralba and Efros showed systematic dataset bias across benchmarks [13]; Recht et al. found 11–14 point drops when ImageNet classifiers were re-evaluated on faithfully reconstructed test sets [10]; Hendrycks and Dietterich measured brittleness to common corruptions [11]; and WILDS showed large gaps on naturally occurring shifts [12]. Geirhos et al. trace much of this brittleness to shortcut learning—reliance on cues that are predictive in the curated distribution but absent in deployment [14]. In tourism vision, lighting, weather, occlusion, and clutter are recognised obstacles [7,32], yet published landmark classifiers are still evaluated mostly on held-out splits of the same collections. Classical landmark benchmarks do contain user-generated imagery [21,33], but a train-on-curated, test-on-social-media protocol for a deployed regional classifier has, to our knowledge, not been reported. We contribute a natural-shift evaluation in which the test distribution is defined by real social media uploads rather than synthetic corruptions.

### 2.3. Ensemble Learning and Enhancement-Based Diversity

Combining diverse predictors improves accuracy and calibration [34], and deep ensembles are among the most reliable methods under dataset shift [35,36]. Diversity can be obtained cheaply from training snapshots [37] or, at test time, by aggregating predictions over transformed views of the input [38]; surveys catalogue the design space [39]. A complementary source of diversity is systematic input transformation: adaptive histogram equalisation [40] and, more recently, pixel-interval power transforms [41], triple-threshold intensity ensembles for microscopy [42], and dual-pathway fusion of raw and enhanced views in medical imaging [43] all show that models trained on differently enhanced variants of the same data learn complementary representations. Our prior work carried this principle into landmark recognition, first with a single squaring threshold [8] and then with a graduated multi-threshold scheme and a validation-driven selective ensemble that discards non-contributing members [9]. What remained untested is whether this intensity-domain diversity—designed for accuracy on curated data—also confers robustness under natural domain shift. That question is central here, and Section 4.2 isolates it with a seed-diversity control.

### 2.4. Multimodal Large Language Models as Zero-Shot Recognisers

Contrastive vision–language pretraining enables zero-shot classification by matching images to textual class descriptions [16], and instruction-tuned frontier systems extend this to open-ended visual question answering [17,18,19,44,45,46]. Early studies of GPT-4V reported strong general scene understanding but unreliable fine-grained identification [18]. Landmark recognition for a specific region stresses exactly this weakness: the categories are visually similar, geographically clustered, and referred to by transliterated names (e.g., Sherdor, Tillya-Kori, Gur-e-Amir) that appear with inconsistent spellings in web corpora. Quantitative evidence on how current frontier MLLMs perform on such under-represented heritage categories is scarce; the preliminary comparison in Section 4.3 provides an initial measurement for Central Asian landmarks and sets it against a specialised supervised alternative.

## 3. Materials and Methods

**Figure 1:**
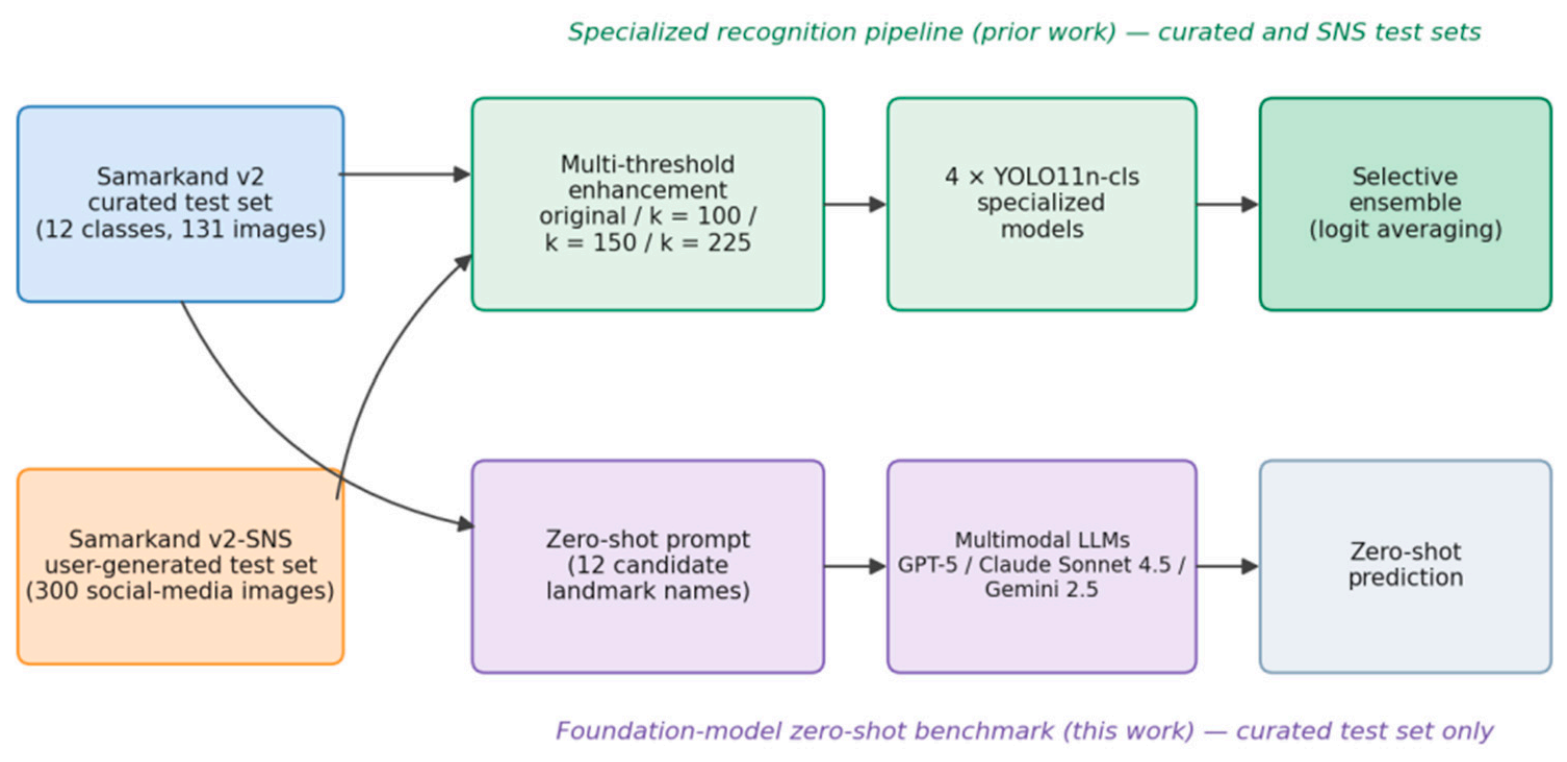
Overview of the evaluation design. The specialised pipeline (multi-threshold enhancement, four YOLO11n-cls models, selective logit-averaging ensemble) follows [8,9] and is trained only on curated Samarkand v2 imagery. Both the curated test split and the user-generated Samarkand v2-SNS set are classified by the specialised systems; the curated test split is additionally classified by three zero-shot MLLMs. Arrows indicate the data flow; the upper row is the specialised recognition pipeline and the lower row the zero-shot MLLM benchmark.

Figure 1 summarises the design. A single query image is classified by two independent systems: the specialised multi-threshold selective ensemble of [9] (upper branch) and, for comparison, three zero-shot MLLMs under a constrained prompt (lower branch). Supervised systems are trained only on curated Samarkand v2 imagery and evaluated both in-distribution (curated test split) and out-of-distribution (Samarkand v2-SNS).

### 3.1. Specialised Recognition Framework

We evaluate the multi-threshold enhancement and selective ensemble of [8,9] exactly as published; no component is retrained or retuned. Given an 8-bit image with pixel intensity *p*(*x*, *y*), the enhancement transform of Equation (1) squares every value above a threshold *k*:

(1) $$I\left(x,y\right)=\{\begin{array}{ll} {\left[p\left(x,y\right)\right]}^{2}, & \mathrm{if}\;p\left(x,y\right)>k\\ p\left(x,y\right), & \mathrm{otherwise} \end{array}\;\;k\in \{100,150,225\}$$

followed by renormalisation to the network input range. The three thresholds emphasise complementary evidence: *k* = 100 amplifies shadowed carvings and fine texture, *k* = 150 mid-tone painted and tiled surfaces, and *k* = 225 reflective glazed and sunlit elements [9]. Four YOLO11n-cls classifiers [29] with identical configurations are trained on the original images (M_O) and on the three enhanced variants (M_E100, M_E150, M_E225). All models use 640 × 640 inputs, the Adam optimiser (initial learning rate 0.001, cosine annealing with 3 warm-up epochs), batch size 16, weight decay 0.0005, cross-entropy loss, early stopping with patience 10 within a 100-epoch budget, horizontal flips and ±10° rotations for augmentation, and a fixed random seed of 42; training used the Ultralytics YOLO11 framework (version 8.3.x) on an NVIDIA RTX 3090 GPU [9].

At inference, an ensemble subset *S* produces logits by arithmetic averaging, *z*(*S*) = (1/|*S*|) Σ *z*(*i*), and the predicted class is the arg max after a single softmax (Algorithm 1). The deployed subset *S\ is chosen once, on the curated validation split, by evaluating all 2^4^ − 1 = 15 non-empty subsets and keeping the most accurate. Paired t-tests in [9] showed that the k = 100 and k = 150 variants improve significantly over the original model, while k = 225 does not. Because subset selection maximises ensemble validation accuracy rather than individual-member significance, the selected subset need not contain the individually strongest members. The subset selected on validation and used for every ensemble result in this paper is S\* = {M_E100, M_E150, M_E225}: the search dropped the model trained on original imagery (M_O) and kept the three enhanced variants, whose complementary errors it exploits. Restricting inference to these three members also reduces ensemble cost by about 25% relative to the full four-member configuration [9]. No information from Samarkand v2-SNS or from any test split was used for subset selection.
**Algorithm 1.** Selective multi-threshold ensemble inference  Input: image *x*; selected member set *S*\* = {M_E100, M_E150, M_E225}; thresholds* k*.  1. For each member *m* ∈ *S*\*: apply the member’s enhancement transform to* x *and compute logits* z_m*.  2. Average the logits: $\bar{z}$ = (1/|*S*\*|) Σ_m* z_m*.  3. Apply a single softmax to $\bar{z}$ and return the arg max class.

### 3.2. Curated Benchmark: Samarkand v2

Samarkand v2 [9] contains photographs of twelve historical landmarks of Samarkand: Al-Buxoriy Mausoleum, Bibi-Khanym Mosque, Gur-e-Amir Mausoleum, Hazrati Doniyor Mausoleum (Tomb of Daniel), Imom Motrudiy Complex, Ruhobod Complex, Sherdor Madrasa, Tillya-Kori Madrasa, Ulugh Beg Observatory, Ulugh Beg Madrasa, Khizr Complex, and the Shah-i-Zinda Necropolis. Images were captured on site from multiple vantage points across seasons, times of day, and weather conditions, annotated with multi-stage expert verification, and resized to 640 × 640 [9]. The public release on Kaggle (https://www.kaggle.com/datasets/ulugbekhudayberdiev/samarkand-v2, accessed on 19 August 2026) comprises 1258 images in fixed training (1122) and validation (136) splits across the twelve classes. The additional held-out curated test set of 131 images, on which all in-distribution results in [9] and in this study are reported, is not part of the public release and is available from the corresponding author upon reasonable request.

### 3.3. Out-of-Distribution Benchmark: Samarkand v2-SNS

To measure generalisation to the imagery a deployed service actually receives, we assembled Samarkand v2-SNS (SNS: social networking service), a test-only set of user-generated photographs of the same twelve landmarks, downloaded in November–December 2025 from publicly accessible social and travel platforms: Instagram, VK, TripAdvisor, and Google Maps user photographs. The collection protocol enforced four rules. (i) *Disjointness:* no image, near-duplicate, or crop of a Samarkand v2 training or validation image was admitted; candidates were screened by perceptual hashing against those splits, and every near-match was resolved by manual side-by-side review. (ii) *Authenticity:* only genuine visitor uploads were kept—no stock photography, official tourism-board imagery, or postcard scans. (iii) *Label reliability:* each image was labelled independently by two annotators familiar with the sites, and only images on which both agreed were admitted; ambiguous images were discarded. Label reliability thus rests on dual-annotator consensus; inter-annotator agreement over the full 360-image candidate pool (before the remaining screening filters) was high—observed agreement 0.94, expected (chance) agreement 0.084, giving Cohen’s κ = 0.93, which is “almost perfect” agreement on the Landis–Koch scale. (iv) *Identifiability:* the landmark had to be recognisable to a human expert; images dominated by people with only incidental background architecture were excluded.

Only publicly accessible content was collected, without circumventing access controls, and no personal data beyond the images were collected or stored. Because copyright remains with the uploaders, the image files are not redistributed publicly (see the Data Availability Statement).

The resulting set contains 300 images, 25 per class. Compared with curated imagery, the SNS photographs show lower and more variable resolution, aggressive JPEG recompression, extreme viewing angles, night-time and indoor illumination, heavy occlusion by crowds, and stylistic filters. No SNS image was used for training, validation, hyperparameter tuning, or subset selection; the set serves purely as a natural distribution-shift probe in the spirit of [10,12].

### 3.4. Zero-Shot Multimodal LLM Evaluation Protocol

Three frontier multimodal systems were evaluated on the curated Samarkand v2 test split: GPT-5 [44], Claude Sonnet 4.5 [45], and Gemini 2.5 [46]. The first author ran the evaluation in late 2025 through each provider’s consumer web-chat interface, using the default production model offered at the time. Because access was interface-mediated, the model names here refer to the consumer-tier defaults of ChatGPT, Claude.ai, and the Gemini app at evaluation time, not to pinned API versions. Consumer interfaces may add undocumented system prompts, dynamic image compression, tool augmentation, and silent model updates between sessions, so the reported accuracies characterise the end-user products rather than the underlying models in isolation; “zero-shot” here means only that no task-specific examples were given.

Each test image was submitted with a constrained instruction asking the model to name which of the twelve Samarkand landmarks is shown and to answer with a single landmark name (Appendix A gives the instruction template). Responses were mapped to class labels manually, allowing transliteration variants (e.g., “Bibi-Khanym” vs. “Bibikhonim”); a response naming no listed landmark was scored as incorrect. Two of the 131 test images could not be processed through the web interfaces and were excluded for all three systems, so every MLLM accuracy here is computed over the remaining 129 images. Because the original per-image logs, exact prompt wording, and interface version identifiers were not archived, the accuracies in Section 4.3 are the tallies recorded at evaluation time, as documented in the first author’s doctoral dissertation [47], and should be read as a preliminary snapshot rather than a fully reproducible benchmark.

We evaluate the MLLMs on the curated split rather than the SNS split so that their scores are directly comparable to the supervised in-distribution results and are not confounded by the domain shift; extending the zero-shot benchmark to the SNS set under a fully logged protocol is left to future work.

### 3.5. Metrics

Supervised systems are scored on the curated split with top-1 accuracy; the macro-averaged precision, recall, and F1-score for these models are reported in [9]. On the exactly class-balanced SNS set (25 images per class), macro-averaged recall equals top-1 accuracy, so we report top-1 accuracy with a Wilson confidence interval as the primary cross-domain metric. MLLMs are scored with top-1 accuracy. For cross-domain analysis, we also report the absolute accuracy drop in percentage points, the relative drop, and the error-rate ratio between the SNS and curated sets. Because both test sets are fixed and every system sees identical samples, differences between systems on the same set are directly comparable. For the 300-image SNS set, we report 95% Wilson score confidence intervals; two systems whose intervals do not overlap are treated as reliably different. This criterion is stricter than a single 5%-level test and conservative relative to a paired McNemar test on identical samples. Section 4.1 additionally reports marginal-count bounds on the exact McNemar statistic that hold under every joint outcome consistent with the reported accuracies.

## 4. Results

### 4.1. Cross-Domain and Per-Class Performance

**Figure 2:**
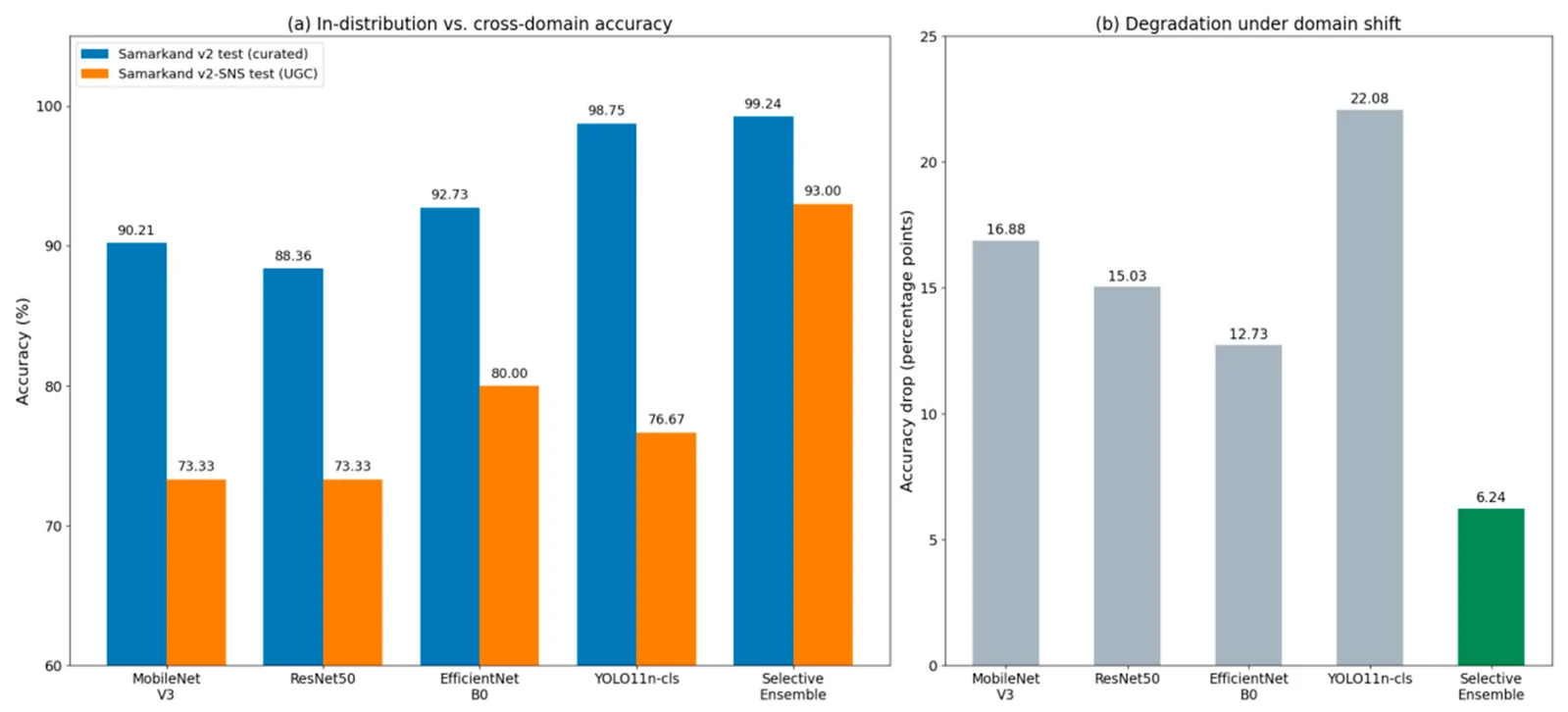
Cross-domain evaluation. (**a**) Accuracy on the curated Samarkand v2 test split (blue) and on Samarkand v2-SNS (orange). (**b**) Absolute accuracy drop under the shift; the selective ensemble (green) degrades roughly one-half to one-quarter as much as the single-model baselines. In (**a**), the vertical axis starts at 60% to resolve differences among systems, with exact values printed above each bar.

Table 1 consolidates the core result: for every system, the curated top-1 accuracy, the SNS top-1 accuracy with its Wilson interval, and the degradation under the shift. The reference in-distribution values are those reported in [9] and are not recomputed here. As printed, they need not correspond to an integer number of correct classifications out of 131 test images, so the error-inflation column should be read as indicative rather than exact. Figure 2 visualises the comparison. Every system loses accuracy under the shift—confirming that curated evaluation overstates deployment performance—but the size of the loss differs sharply across systems.

Three points stand out. First, the four baselines drop by 12.73 to 22.08 points, landing in the 73–80% range—a 20–27% error rate, or roughly one misidentified photograph in every four to five, a level unlikely to sustain a user-facing service. Second, the in-distribution ranking does not hold: YOLO11n-cls, the strongest baseline on curated data by six points over EfficientNetB0, falls behind it on UGC (76.67% vs. 80.00%, a 10-image gap whose intervals overlap) and suffers the largest absolute drop of all systems (−22.08 points); its curated error rate of 1.25% multiplies by a factor of 18.7 to 23.33%. This pattern is consistent with shortcut-like reliance on distribution-specific cues [14]. Third, the selective ensemble is by far the most robust system: it loses only 6.24 points (99.24% to 93.00%) and beats the strongest baseline on UGC by 13.00 points—a 65% relative reduction in error rate (7.00% vs. 20.00%).

The ensemble’s advantage on UGC is statistically reliable, not an artefact of the finite test set. Its interval (89.5–95.4%) does not overlap that of EfficientNetB0 (75.1–84.1%). A paired analysis is possible even without the per-image records: because all systems classify the identical 300 images, the discordant counts *b* (ensemble correct, EfficientNetB0 wrong) and *c* (the converse) must satisfy *b* − *c* = 39 and *c* ≤ 21, so an exact two-sided McNemar test rejects equality at *p* < 10^−4^ under every joint outcome consistent with the observed marginals (279/300 vs. 240/300); the corresponding bound against each of the other three baselines is *p* < 10^−6^. No such unconditional statement holds for the EfficientNetB0–YOLO11n-cls reversal, whose 10-image margin admits outcomes on either side of the 5% level; we therefore treat that reversal as suggestive rather than established.

The ensemble does not eliminate the gap. Its error rate also rises under the shift, from 0.76% to 7.00%—a 9.2-fold increase—so the domain gap is mitigated, not closed. What matters in practice is the absolute level it sustains on UGC (93.00%, fewer than one error in 14 photographs) and its margin over every individual architecture evaluated under identical conditions.

**Per-class failure modes.** Table 2 breaks the UGC accuracy down by landmark for the selective ensemble and the strongest baseline (EfficientNetB0). Two patterns stand out. First, the ensemble outperforms the baseline on every one of the 12 classes, so its robustness advantage is uniform rather than driven by a few categories. Second, the errors of both systems concentrate in the visually less distinctive complexes—Ruhobod Complex (84.0% ensemble, 68.0% baseline), Imom Motrudiy Complex (88.0%/72.0%), and Ulugh Beg Madrasa (88.0%/68.0%)—whereas the most iconic, visually singular structures, Sherdor Madrasa and the Ulugh Beg Observatory, are recognised almost perfectly (100.0%/92.0% each). The ensemble’s largest gains fall precisely on the hardest classes (for example, Tillya-Kori Madrasa and Ulugh Beg Madrasa, every +20 points over the baseline), so the multi-threshold enhancement helps most where a single model is least reliable. This per-class pattern is consistent with the shortcut-learning interpretation of Section 5.2: recognition that leans on a few distinctive cues survives the shift for singular monuments but degrades for the repetitive glazed-tile complexes whose discriminative detail is washed out by social-media compression and lighting.

**Figure 3:**
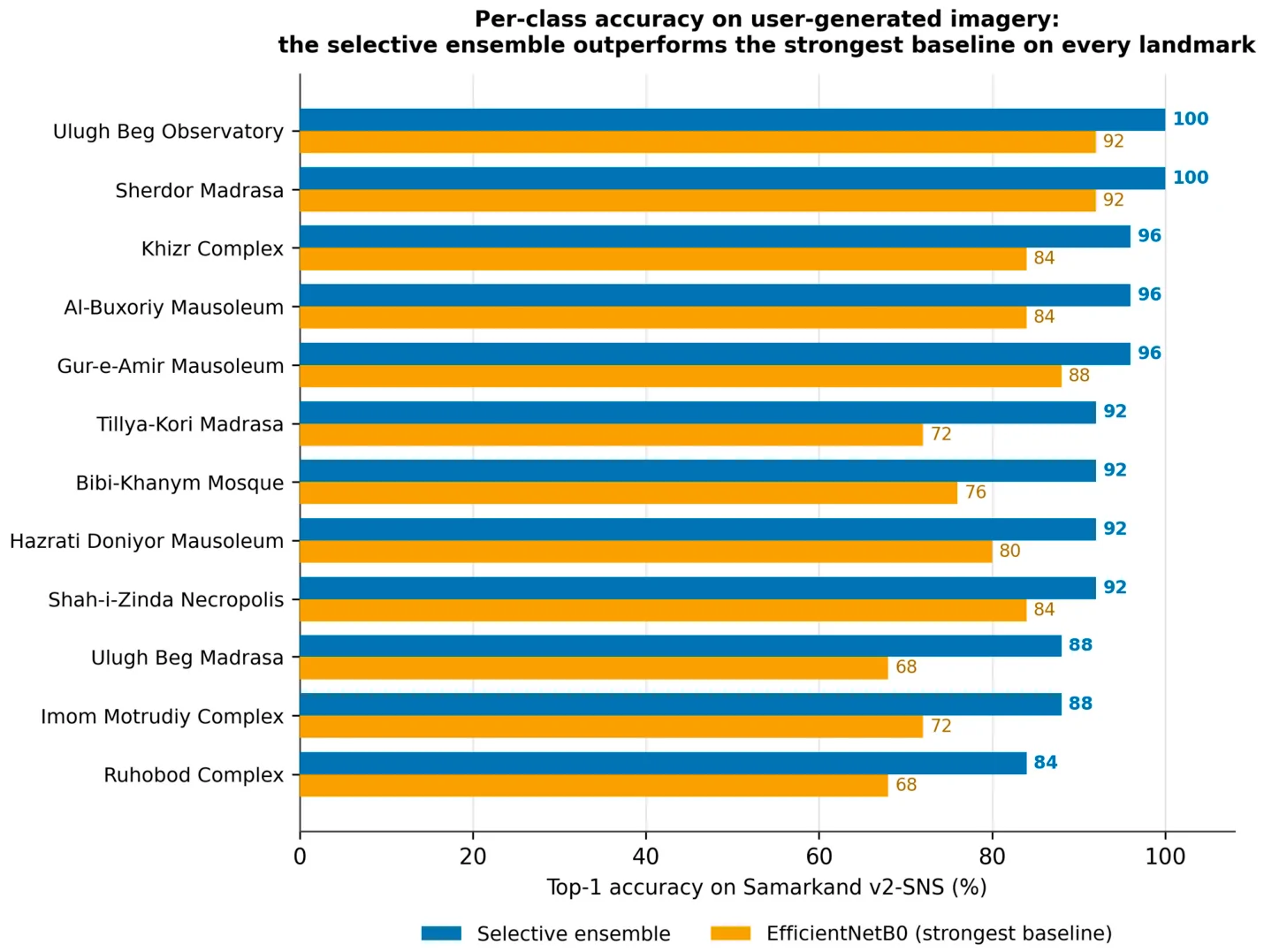
Per-class top-1 accuracy on the Samarkand v2-SNS user-generated test set (25 images per class): the selective ensemble (blue) vs. the strongest single-model baseline, EfficientNetB0 (orange). The ensemble improves accuracy on every one of the 12 landmark classes (overall 93.0% vs. 80.0%; Table 2); classes are ordered by ensemble accuracy.

Figure 3 visualises this per-class comparison: the selective ensemble’s accuracy exceeds the strongest baseline’s for every one of the twelve landmarks, and its largest margins fall on the hardest classes.

**Figure 4:**
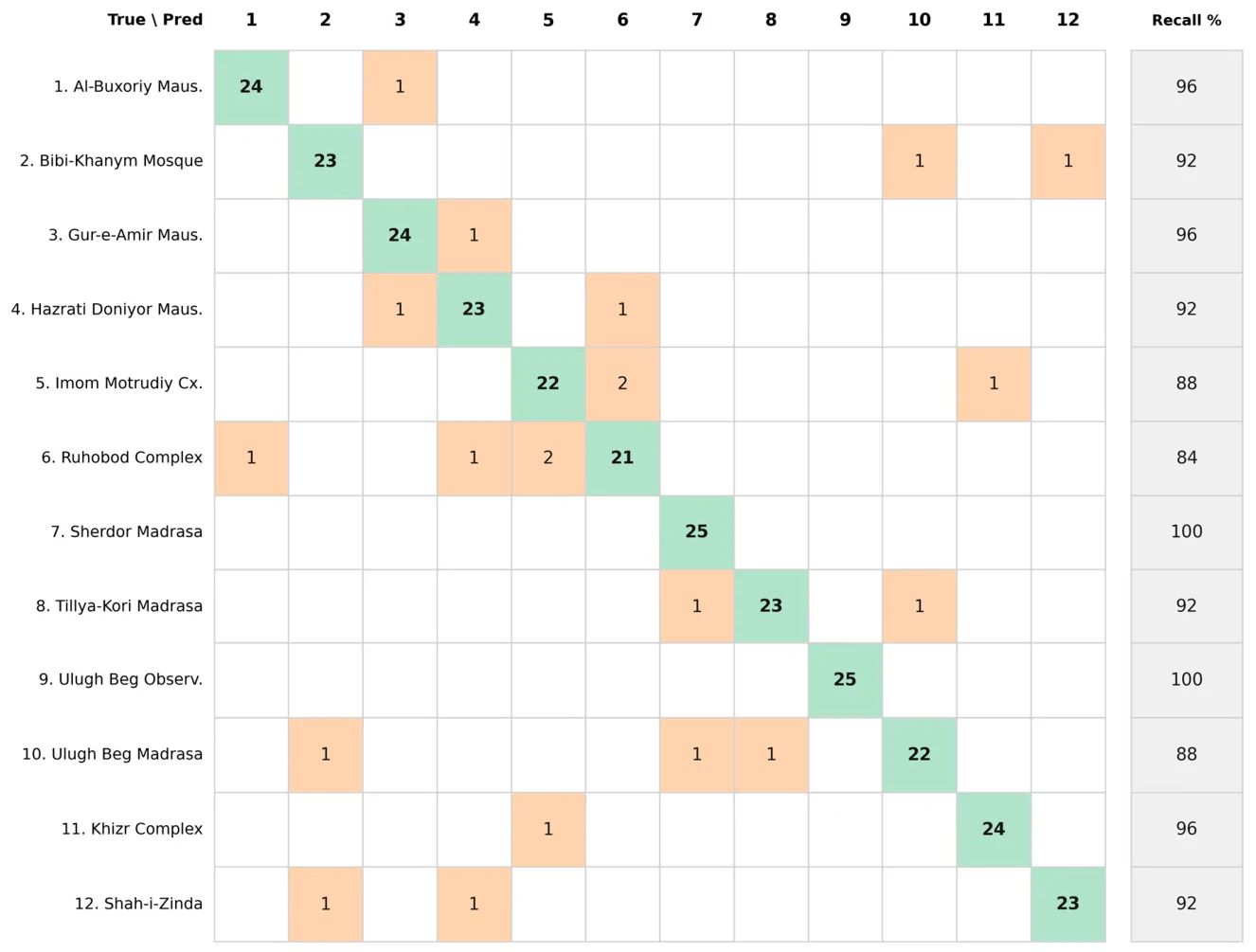
Confusion matrix of the selective ensemble on Samarkand v2-SNS (rows = true class; columns = predicted class 1–12; green = correct on the diagonal; amber = misclassification; 25 images per class, with per-class recall at right). Residual errors fall predominantly among visually similar monuments—the Registan madrasas (7, 8, 10), the mausoleums (1, 3, 4), and the smaller tiled complexes (5, 6, 11)—rather than across dissimilar architectural types.

The full confusion matrix (Figure 4) shows where the residual errors go. They fall predominantly among visually similar monuments: the three Registan madrasas (Sherdor, Tillya-Kori, Ulugh Beg Madrasa) are confused with one another, the mausoleums (Al-Buxoriy, Gur-e-Amir, Hazrati Doniyor) with one another, and the smaller tiled complexes (Imom Motrudiy, Ruhobod, Khizr) with one another; confusions across dissimilar architectural types are few (for example, Bibi-Khanym Mosque with Ulugh Beg Madrasa). This concentration of errors among the most look-alike façades—rather than a uniform spread—reinforces the shortcut-learning reading of Section 5.2: what survives the shift is the coarse, family-level appearance rather than the fine ornamental detail that separates near-identical Timurid monuments.

**Figure 5:**
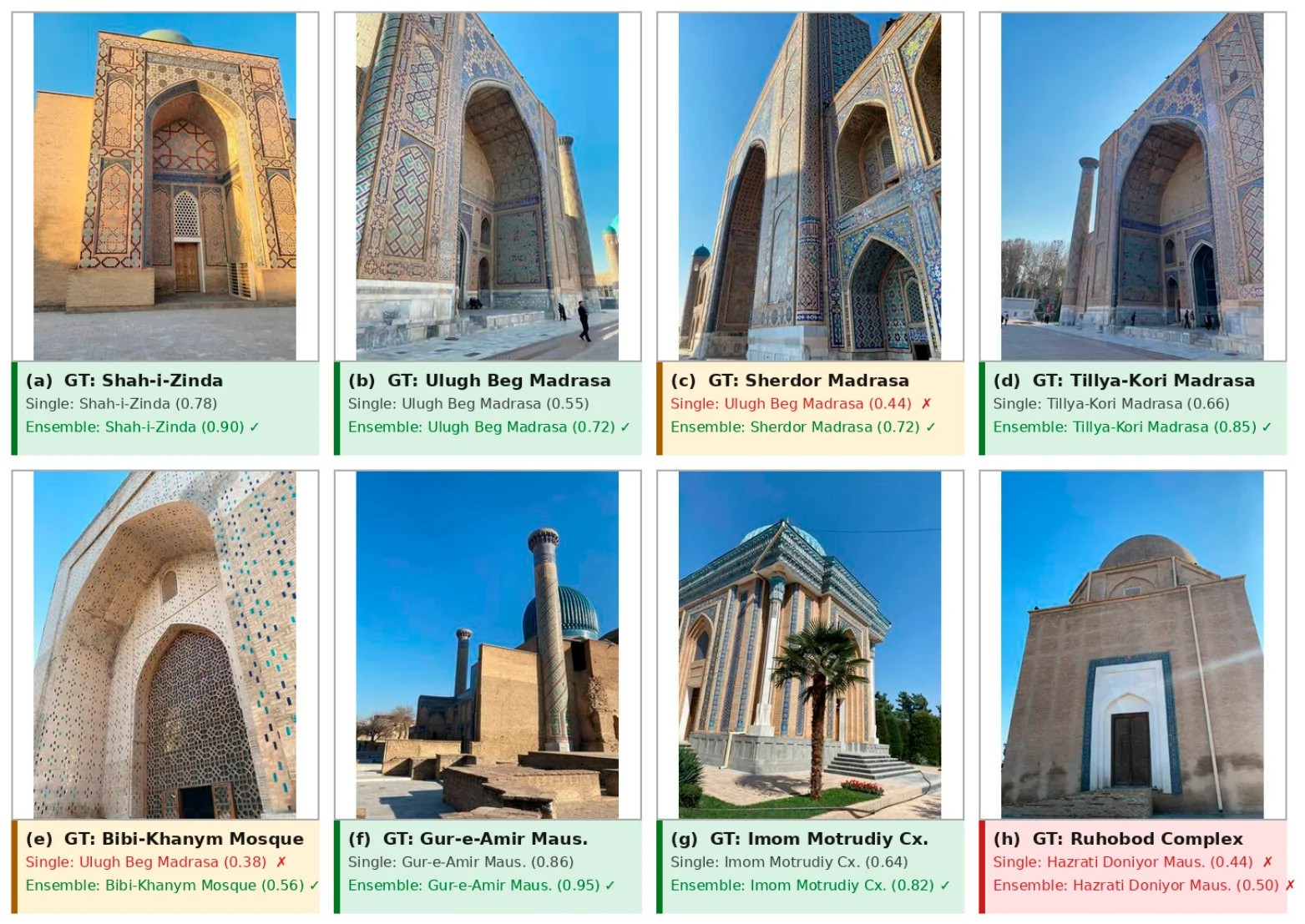
Qualitative cross-domain analysis on Samarkand v2-SNS: the single model (M_O) vs. the selective ensemble on eight user-generated photographs (one landmark each) (**a**–**h**). Each panel gives the ground-truth class and the predicted class with confidence for the single original-image model and for the ensemble (✓ = correct, ✗ = incorrect). Strip colour encodes the outcome: green = both systems correct; amber = the single model fails, but the ensemble recovers (**c**,**e**); red = the ensemble also fails (**h**). Per-member predictions (M_O, M_E100, M_E150, M_E225) for every panel are listed in Appendix B, Table A1.

To make the cross-domain behaviour concrete at the level of individual photographs, Figure 5 presents a qualitative gallery of eight user-generated images—one landmark each, spanning 8 of the 12 classes—annotated with the ground-truth label and the predictions, with confidence, of the single original-image model (M_O, the standalone YOLO11n-cls baseline) and of the selective ensemble. Three behaviours are visible. In the green panels, both systems are correct, though the ensemble is the more confident (for example, Shah-i-Zinda rises from 0.78 to 0.90 in panel (a)). The two amber panels are exactly the ensemble-corrects-single-model cases behind the aggregate gain of Table 3: on a steep upward view of Sherdor Madrasa (c) the single model picks a sister Registan madrasa (Ulugh Beg Madrasa, 0.44) while the enhancement members agree on the correct class (0.72), and on an extreme partial-view crop of the Bibi-Khanym Mosque (e) the single model again fails (Ulugh Beg Madrasa, 0.38) while the ensemble recovers (0.56). The single red panel (h) is an honest failure: a plain, backlit brick façade of the Ruhobod Complex—one of the least distinctive monuments—is confused with another plain brick mausoleum (Hazrati Doniyor), which is the one such error in the confusion matrix (Figure 4). The complete per-member predictions for all eight panels are given in Appendix B (Table A1).

**Figure 6:**
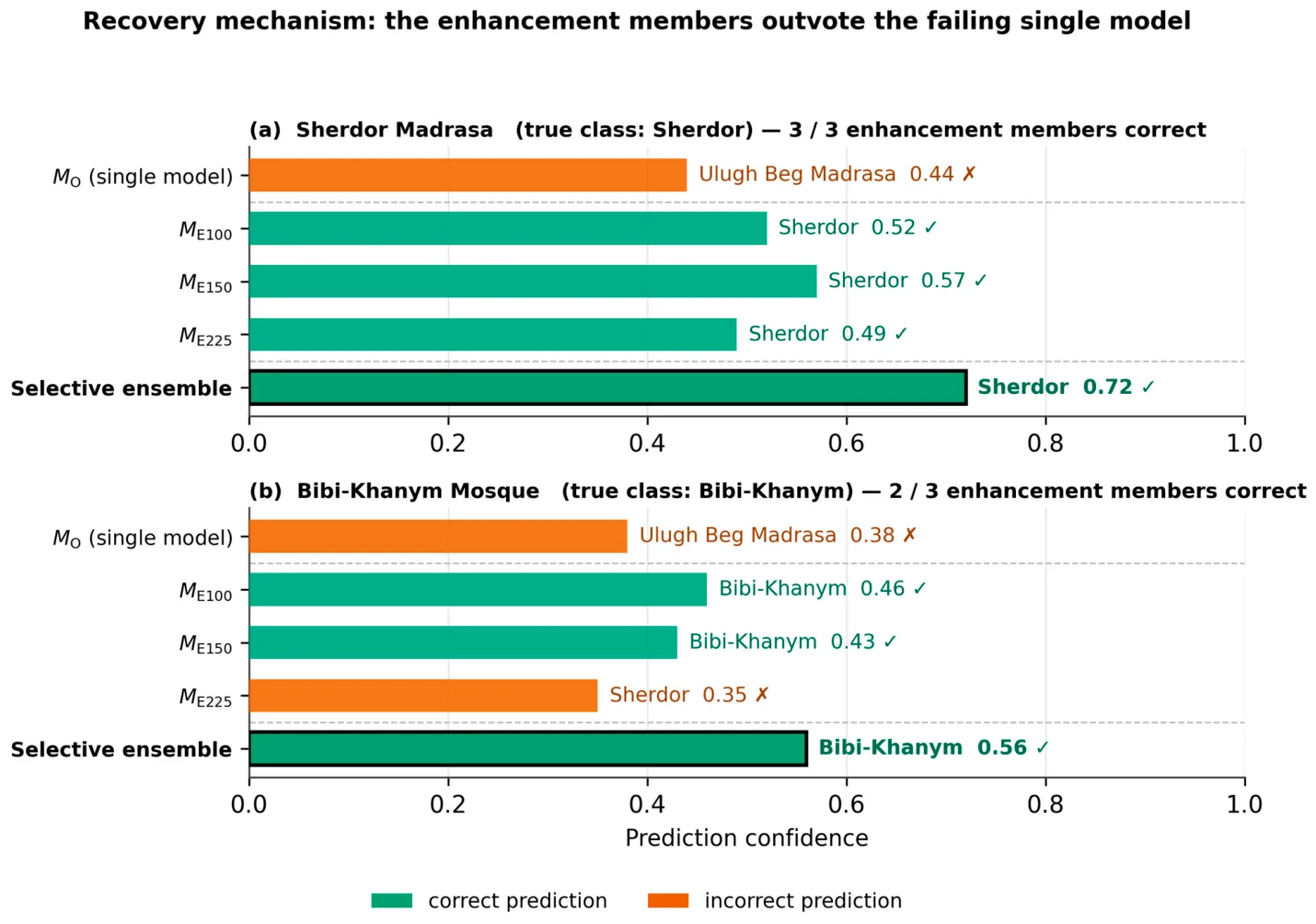
Per-member prediction confidences for the two recovery cases of the qualitative gallery (Figure 5c,e; full values in Appendix B, Table A1) (**a**,**b**). In each case, the single original-image model M_O misclassifies the user-generated photograph (orange), while the three enhancement members M_E100, M_E150, M_E225—the voters of the selective ensemble—predict the correct class (green), so the logit-averaged ensemble decision (outlined, bottom) recovers the correct landmark. For Sherdor Madrasa, all three members agree; for the harder Bibi-Khanym Mosque crop, two of three suffice. Dashed rules separate the single original-image model, the three enhancement members, and the selective-ensemble decision. ✓ = correct, ✗ = incorrect.

Figure 6 makes this recovery mechanism explicit for the two amber cases. The single original-image model M_O predicts the wrong landmark, whereas the three enhancement members—the voters of the selective ensemble—predict the correct class (all three for Sherdor Madrasa, two of three for the harder Bibi-Khanym Mosque crop), so the logit-averaged decision recovers it.

A note on fairness is warranted. The ensemble aggregates three networks, whereas each baseline is a single model, so the comparison is between deployable systems rather than parameter-matched architectures. The capacity gap is nonetheless small: the deployed three-member subset totals about 4.64 million parameters, and even the full four-member configuration (about 6.2 million) is of the same order as a single EfficientNetB0 (about 5.3 million) and far below ResNet50 (about 25.6 million). Robustness therefore cannot be explained by parameter count alone. Section 4.2 goes further and isolates the specific contribution of enhancement diversity with a matched seed-diversity control.

### 4.2. Seed-Diversity Ablation

A natural concern is that the ensemble’s robustness is simply the generic benefit of deep ensembling [35,36] rather than a property of enhancement diversity. To separate the two, we compare the deployed enhancement-diverse ensemble against a capacity-matched control: a seed-diverse ensemble of three YOLO11n-cls models trained on the raw (unenhanced) imagery with different random seeds (42, 43, 44) and fused by the same logit averaging. Both ensembles have three members and about 4.64 million parameters, so they differ only in their source of diversity—random initialisation vs. multi-threshold enhancement. Table 3 reports the result.

In-distribution, all three configurations sit at the ceiling (98.47–99.24%), and the differences are within a single image. The informative comparison is on UGC. Seed diversity alone lifts accuracy from 76.67% to 82.00% (+5.33 points)—a real but modest gain, consistent with the known robustness benefit of deep ensembles. Enhancement diversity lifts it further, from 82.00% to 93.00% (+11.00 points). Enhancement diversity therefore accounts for roughly two-thirds of the total improvement over the single model, and about twice as much as generic seed diversity. This directly addresses the confound: the ensemble’s robustness is driven mainly by the multi-threshold enhancement, not by the mere fact of averaging several models.

### 4.3. Preliminary Zero-Shot Multimodal LLM Comparison

**Figure 7:**
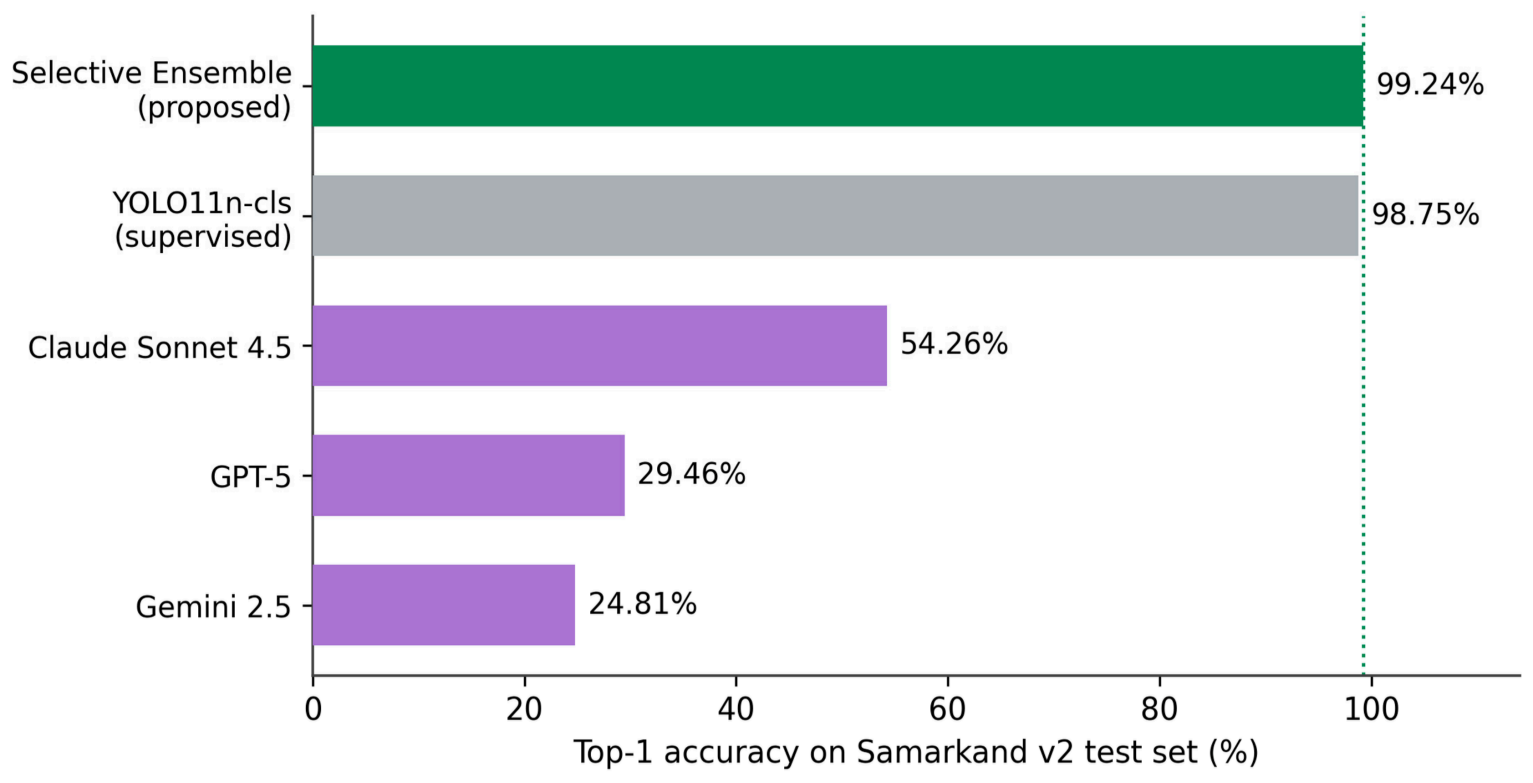
Zero-shot MLLMs vs. supervised specialised systems on the curated Samarkand v2 test split (top-1 accuracy; MLLMs on 129 of 131 images, Section 3.4). The selective ensemble is the multi-threshold model proposed in [9]. The dotted line marks the selective ensemble (99.24%). Bars are coloured by system type: green for the proposed selective ensemble, grey for the supervised YOLO11n-cls baseline, and purple for the three zero-shot MLLMs.

Because these evaluations were run through consumer web interfaces without pinned model versions or archived per-image logs (Section 3.4), we report them as a preliminary, qualitative exploratory baseline rather than as a verifiable benchmark: the comparison is meant to bound current MLLM capability on this task, not to rank specific model versions. Table 4 and Figure 7 report the zero-shot accuracy of the three MLLMs on the curated Samarkand v2 test split (129 of 131 images; Section 3.4), alongside the supervised references. Claude Sonnet 4.5 is the strongest foundation model at 54.26% (70/129; 95% Wilson CI 45.7–62.6%), followed by GPT-5 at 29.46% (38/129; 22.3–37.8%) and Gemini 2.5 at 24.81% (32/129; 18.2–32.9%). All three sit well above the 8.33% chance floor of the twelve-way protocol; Claude’s lead is interval-separated from the other two, while the GPT-5–Gemini ordering is not resolved at this sample size. Even the best MLLM remains about 45 points below the selective ensemble (99.24%, on the full 131-image split) and below every supervised baseline, including the weakest (ResNet50, 88.36%). All three MLLMs also score below the specialised systems’ out-of-distribution accuracy on UGC (Table 1), so the gap cannot be attributed to test-set difficulty.

Because two of the 131 curated test images could not be processed through the web interfaces (Section 3.4), the accuracies above are computed over the remaining 129. For an even-handed comparison with the supervised systems, which are scored on all 131 images, we also recompute the MLLM accuracies over the full 131-image split, counting each unprocessable upload strictly as a classification failure (Table 5). The effect is negligible—Claude Sonnet 4.5 53.44%, GPT-5 29.01%, Gemini 2.5 24.43%—and the ranking and every conclusion are unchanged.

The size of the gap is consistent with the under-representation of transliterated Central Asian landmark names and imagery in web-scale pretraining; Section 5.3 examines this coverage gap and its implications. A per-image failure-mode analysis is not reported because the original evaluation logs were not retained (Section 3.4); a fully logged replication is identified as future work.

### 4.4. Synthesis

Taken together, the experiments locate the current division of labour. Specialised supervised pipelines remain necessary for fine-grained regional heritage recognition: zero-shot foundation models are 45–74 points short of deployment accuracy. Within the specialised family, curated-benchmark accuracy is an unreliable proxy for field performance—the single best curated-split model loses its advantage entirely under natural shift—whereas enhancement-diverse selective ensembling transfers its advantage across domains at a parameter budget of the same order as a single efficient backbone, and the ablation of Section 4.2 attributes most of that transferred advantage to enhancement diversity rather than to ensembling per se.

## 5. Discussion

### 5.1. Why Does Intensity-Domain Diversity Confer Robustness?

The selective ensemble was designed in [9] to maximise accuracy on curated data, yet its largest measured benefit is robustness under natural shift, and Section 4.2 shows this benefit is specific to enhancement diversity. Three mechanisms plausibly combine. First, the multi-threshold transform gives each member a systematically different view of the same scene: the *k* = 100 member anchors its decisions in shadow-region texture, while the *k* = 150 member relies on mid-tone tilework. A perturbation that breaks one cue family—an overexposed night shot, filtered colours, crushed shadows in a compressed upload—does not break the others, so logit averaging suppresses errors that are uncorrelated across intensity domains. This is the classical diversity argument for ensembles [34,35], instantiated in the input-transformation space rather than in weights or architectures, and it parallels the benefits reported for aggregating transformed views at test time [38] and for dual-pathway raw-plus-enhanced designs [41,42,43]. Second, deep ensembles degrade more gracefully than single models as shift increases [36]; our results extend this from synthetic corruptions to a real UGC shift. Third, the selective subset search removes members whose contribution is not supported by validation evidence [9], preventing a systematically unhelpful view from adding correlated noise when conditions are hardest. Critically, the seed-diversity control in Section 4.2 shows that these effects are not merely the generic benefit of averaging several models: a matched seed-diverse ensemble recovers only part of the robustness, so the multi-threshold enhancement is doing most of the work.

### 5.2. Curated Rankings Are Not Deployment Rankings

The reversal between YOLO11n-cls and EfficientNetB0—six points apart in one direction on curated data, three points apart in the other on UGC—carries a methodological lesson for applied landmark recognition: model selection performed only on a curated validation split can pick precisely the model that generalises worst. The 18.7-fold error inflation of YOLO11n-cls suggests it fit distribution-specific regularities of the curated protocol (framing conventions, capture equipment, favourable vantage points), a behaviour consistent with shortcut learning [14] and with benchmark-overfitting on ImageNet-scale evaluations [10,13]. We therefore recommend that studies proposing landmark classifiers for smart tourism report at least one natural out-of-distribution measurement—social-media imagery being the most faithful to deployment—alongside curated metrics.

### 5.3. Implications of the Foundation-Model Gap

The 45–74 point deficit of zero-shot MLLMs is unlikely to reflect a lack of general visual competence. The most plausible explanation—consistent with, though not demonstrated by, our data—is that fine-grained regional heritage sits in a coverage gap of web-scale pretraining. The 12 Samarkand categories are visually similar variations of a shared architectural language, are documented online far less densely than Western European landmarks, and carry transliterated names with unstable spellings. Claude Sonnet 4.5’s clearly stronger result (54.26%) shows that frontier systems are beginning to accumulate such knowledge, but none approaches the reliability a tourist-facing service requires. The architecture that follows from these numbers is hybrid: a compact on-device recogniser establishes what the monument is with 93–99% reliability, and an MLLM consumes the predicted label to generate multilingual narration, historical context, and accessibility descriptions—capabilities the specialised model entirely lacks. Because the recogniser still errs on roughly 1 in 14 UGC images, the hand-off should propagate the ensemble’s confidence and abstain below a threshold, so that residual recognition errors are not amplified into fluent but incorrect narration. This division also keeps MLLM inference cost and latency off the recognition path, preserving offline operation. Keeping recognition on-device is a privacy advantage in itself, since user photographs need not leave the phone; where images must nonetheless be stored or transmitted, privacy-preserving techniques such as image encryption [48] are a complementary safeguard for a production smart-tourism service that continuously ingests user imagery. The specialised path is compact enough for the role: each YOLO11n-cls member has about 1.55 million parameters [29], so even the full four-member ensemble (about 6.2 million) remains smaller than a single ResNet50 (about 25.6 million).

Beyond parameter count, Table 6 reports measured deployment efficiency. A single member runs in 19.9 ms per image on a T4 GPU (about 50 FPS) and 89.2 ms on CPU; the deployed three-member ensemble, which performs three forward passes, runs in 65.7 ms on GPU (about 15 FPS) and 267.3 ms on CPU, at a total model size of 9.2 MB. For the target use case—a tourist capturing a single photograph rather than processing a video stream—15 FPS on a modest GPU and a CPU latency of about 0.27 s are more than adequate, and because the three members are independent, they can be batched or run in parallel to approach single-model latency. At 9.2 MB and 4.64 M parameters, the pipeline sits well within the envelope of on-device mobile deployment through NCNN or TFLite export; a continuous real-time video setting, if ever required, would fall back to the single strongest member.

### 5.4. Limitations

Five limitations bound the conclusions. First, Samarkand v2-SNS is modest in size (300 images), covers a single city and architectural tradition, and—by construction—does not reproduce two conditions of real deployment streams. (i) *Long tail:* the set is exactly balanced at 25 images per class, whereas real uploads are dominated by a few popular monuments (for example, the Registan ensemble) and are sparse for lesser-known sites; the reported 93.00% is therefore a balanced-class (macro) estimate, and frequency-weighted accuracy on a long-tailed stream could differ, most plausibly falling on the rarest classes. (ii) *Open set:* the pipeline is a closed-set twelve-way classifier, so a landmark-free photograph or a monument outside the twelve-class inventory is necessarily forced into one of the twelve classes and counted as a false positive. Deployment therefore requires an explicit open-set rejection mechanism—most naturally the confidence-thresholded abstention described for the recognizer-to-MLLM hand-off in Section 5.3—together with evaluation on long-tailed and out-of-inventory imagery; we identify both as necessary next steps. The robustness advantage of enhancement-diverse ensembling should also be replicated on other regions and styles before it is treated as general, and the screening pipeline was not otherwise fully characterised quantitatively (no calibrated perceptual-hash threshold was recorded), although inter-annotator agreement is now reported (Section 3.3; Cohen’s κ = 0.93).

Second, the MLLM benchmark uses a single constrained instruction, one query per image, and no in-context examples; richer prompting, retrieval augmentation, or fine-tuning would likely raise MLLM scores, so our figures characterise the plain zero-shot regime only.

Third, commercial MLLMs evolve; the evaluations were run through consumer web interfaces without pinned versions, and the original per-image logs were not archived (Section 3.4). The numbers are a snapshot of late-2025 behaviour, documented in [47], and may not reproduce exactly against later revisions. A rigorous re-evaluation would instead use deterministic API endpoints (temperature 0, fixed and archived system prompts, and archived per-image outputs that enable error analysis), which we leave to future work.

Fourth, the MLLMs were benchmarked only on the curated split; their behaviour on UGC, and a human expert baseline for both splits, remain open.

Fifth, the seed-diversity ablation of Section 4.2 uses raw imagery and a single alternative seed triple; a fuller factorial over seeds and enhancement settings would tighten the attribution, though the present control already separates enhancement diversity from the generic ensemble effect.

## 6. Conclusions

This study asked whether a specialised landmark-recognition pipeline that is near-perfect on curated benchmarks stays useful against two realities of deployment: user-generated imagery and the availability of general-purpose multimodal foundation models. For the specialised pipeline, both answers are affirmative. Under a natural curated-to-social-media shift on twelve Samarkand landmarks, the multi-threshold selective YOLO11 ensemble of [9] degrades by only 6.24 points (99.24% → 93.00%), while single-model baselines lose 12.73–22.08 points and fall to 73–80%; the ensemble cuts the strongest baseline’s error rate by 65% relative. A capacity-matched ablation attributes most of this robustness to enhancement diversity rather than to generic ensembling. The in-distribution ranking of architectures does not survive the shift, which argues for making out-of-distribution evaluation a standard reporting requirement in smart tourism vision. In a preliminary comparison, zero-shot frontier MLLMs (GPT-5, Claude Sonnet 4.5, Gemini 2.5) reach only 24.81–54.26% under a constrained single-prompt protocol (Section 3.4), suggesting that off-the-shelf foundation models cannot yet support fine-grained recognition of these Central Asian landmarks and are better deployed downstream for interpretation and narration; whether CLIP-style zero-shot matching, lightweight adaptation, or retrieval against a labelled gallery narrows this gap is open. Future work will extend the UGC benchmark to additional regions and traditions, evaluate MLLMs on the SNS split under a fully logged, deterministic API-based protocol (temperature 0, explicit archived system prompts, and archived per-image outputs), add a human expert baseline, and explore privacy- and copyright-compliant mechanisms for sharing Samarkand v2-SNS.

## Figures and Tables

**Table 1:** Cross-domain accuracy of all evaluated models (curated Samarkand v2 test, N = 131 → Samarkand v2-SNS, N = 300). Δ (pp) is the absolute accuracy drop; relative drop is Δ divided by curated accuracy; error inflation is the SNS error rate divided by the curated error rate. Curated values are reproduced from [9].

Model	Curated Top-1 (%)	SNS Top-1 (%)	SNS 95% Wilson CI	Δ (pp)	Rel. Drop (%)	Error Infl. (×)
MobileNetV3	90.21	73.33	68.1–78.0	−16.88	18.7	2.7
ResNet50	88.36	73.33	68.1–78.0	−15.03	17.0	2.3
EfficientNetB0	92.73	80.00	75.1–84.1	−12.73	13.7	2.8
YOLO11n-cls (single)	98.75	76.67	71.6–81.1	−22.08	22.4	18.7
**Selective ensemble (proposed)**	**99.24**	**93.00**	**89.5–95.4**	**−6.24**	**6.3**	**9.2**

**Table 2:** Per-class accuracy on the Samarkand v2-SNS user-generated test set (25 images per class).

#	Landmark Class	*N*	Selective Ensemble (%)	EfficientNetB0 (%)
1	Al-Buxoriy Mausoleum	25	24/25 (96.0)	21/25 (84.0)
2	Bibi-Khanym Mosque	25	23/25 (92.0)	19/25 (76.0)
3	Gur-e-Amir Mausoleum	25	24/25 (96.0)	22/25 (88.0)
4	Hazrati Doniyor Mausoleum	25	23/25 (92.0)	20/25 (80.0)
5	Imom Motrudiy Complex	25	22/25 (88.0)	18/25 (72.0)
6	Ruhobod Complex	25	21/25 (84.0)	17/25 (68.0)
7	Sherdor Madrasa	25	25/25 (100.0)	23/25 (92.0)
8	Tillya-Kori Madrasa	25	23/25 (92.0)	18/25 (72.0)
9	Ulugh Beg Observatory	25	25/25 (100.0)	23/25 (92.0)
10	Ulugh Beg Madrasa	25	22/25 (88.0)	17/25 (68.0)
11	Khizr Complex	25	24/25 (96.0)	21/25 (84.0)
12	Shah-i-Zinda Necropolis	25	23/25 (92.0)	21/25 (84.0)
	**Overall**	300	**279/300 (93.00)**	**240/300 (80.00)**

**Table 3:** Seed-diversity ablation, capacity-matched. Both ensembles have three members and equal parameter budgets; they differ only in the source of diversity.

Configuration	Members	Params (M)	Curated Top-1 (%)	SNS Top-1 (%)
Single YOLO11n-cls (raw, seed 42)	1	1.55	98.75	76.67
Seed-diverse ensemble (raw; seeds 42/43/44)	3	4.64	98.47	82.00
**Enhancement-diverse selective ensemble (proposed)**	3	4.64	**99.24**	**93.00**

**Table 4:** Zero-shot accuracy of multimodal LLMs on the curated Samarkand v2 test split (129 of 131 images; Section 3.4), with supervised systems (full 131-image split) shown for reference.

System	Type	Top-1 Accuracy (%)
Gemini 2.5	Zero-shot MLLM	24.81
GPT-5	Zero-shot MLLM	29.46
Claude Sonnet 4.5	Zero-shot MLLM	54.26
YOLO11n-cls	Supervised	98.75
**Selective ensemble (proposed)**	Supervised	**99.24**

**Table 5:** Zero-shot MLLM accuracy on the curated split under two denominators: the original *N* = 129 (two unprocessable uploads excluded) and the full *N* = 131 (the two unprocessable uploads counted as classification failures).

Model	Correct/129	Top-1% (*N* = 129)	Correct/131	Top-1% (*N* = 131)
Claude Sonnet 4.5	70/129	54.26	70/131	53.44
GPT-5	38/129	29.46	38/131	29.01
Gemini 2.5	32/129	24.81	32/131	24.43

**Table 6:** Deployment efficiency on a single T4 GPU and CPU (batch = 1640 × 640 input). GPU latency is mean ± standard deviation over 300 iterations.

System	Params (M)	Model Size (MB)	GPU Latency (ms/img)	CPU Latency (ms/img)	GPU Throughput (FPS)
Single YOLO11n-cls member	1.55	3.1	19.9 ± 1.3	89.2	≈50
**Selective ensemble (M = 3)**	**4.64**	**9.2**	**65.7 ± 3.3**	**267.3**	**≈15**

## Data Availability

The public release of the Samarkand v2 dataset (training and validation splits: 1122 and 136 images) is openly available on Kaggle at https://www.kaggle.com/datasets/ulugbekhudayberdiev/samarkand-v2 (accessed on 19 August 2026); training code is available at https://www.kaggle.com/code/ulugbekhudayberdiev/train/notebook (accessed on 19 August 2026). The curated test split (131 images) is available from the corresponding author on reasonable request. The Samarkand v2-SNS test set consists of user-generated photographs that remain the property of their uploaders and is therefore not redistributed publicly; access for verification is available from the corresponding author on reasonable request for non-commercial research purposes. Per-image prediction records for all supervised systems on both test sets are available from the corresponding author on reasonable request, enabling exact recomputation of all reported metrics.

## Appendix A. Zero-Shot MLLM Prompt

Each curated test image was submitted to the model in a single turn, with no task-specific examples (zero-shot), using a constrained twelve-way instruction of the following form:

“This photograph shows one of these twelve historical landmarks in Samarkand, Uzbekistan: (1) Al-Buxoriy Mausoleum, (2) Bibi-Khanym Mosque, (3) Gur-e-Amir Mausoleum, (4) Hazrati Doniyor Mausoleum, (5) Imom Motrudiy Complex, (6) Ruhobod Complex, (7) Sherdor Madrasa, (8) Tillya-Kori Madrasa, (9) Ulugh Beg Observatory, (10) Ulugh Beg Madrasa, (11) Khizr Complex, (12) Shah-i-Zinda Necropolis. Identify which one it is and reply with a single landmark name only.”

Responses were mapped to class labels manually, tolerating transliteration variants (e.g., “Bibi-Khanym” vs. “Bibikhonim”); a response naming no listed landmark was scored as incorrect. As noted in Section 3.4, the exact per-image wording and the interface version identifiers were not archived; the template above documents the form of the instruction used. A fully version-controlled re-evaluation via deterministic API endpoints (temperature 0), with archived prompts and per-image outputs, is specified as future work (Section 5.4 and Section 6).

## Appendix B. Per-Image Qualitative Analysis

Table A1 lists the full per-model predictions behind the qualitative gallery in Figure 5, for the eight representative Samarkand v2-SNS photographs (one landmark each). For every panel, it gives the predicted class and confidence of the single original-image model (M_O, the standalone YOLO11n-cls baseline) and of the three enhancement members (M_E100, M_E150, M_E225); the deployed selective ensemble averages the logits of the three enhancement members only (Section 3.1), so M_O is shown for comparison and is not part of the ensemble vote. The two amber panels (c, e) are cases in which the single model fails but the ensemble recovers, and panel (h) is an honest failure in which the one correct enhancement member is outvoted—the single Ruhobod → Hazrati Doniyor error is also visible in the confusion matrix (Figure 4).

**Table A1:** Per-member predictions for the eight panels of Figure 5. Each entry is the predicted class index with its confidence; ✗ marks an incorrect prediction and ✓ a correct ensemble decision. Class indices follow Figure 4: 1 = Al-Buxoriy Mausoleum, 2 = Bibi-Khanym Mosque, 3 = Gur-e-Amir Mausoleum, 4 = Hazrati Doniyor Mausoleum, 5 = Imom Motrudiy Complex, 6 = Ruhobod Complex, 7 = Sherdor Madrasa, 8 = Tillya-Kori Madrasa, 9 = Ulugh Beg Observatory, 10 = Ulugh Beg Madrasa, 11 = Khizr Complex, 12 = Shah-i-Zinda Necropolis.

Panel	Ground Truth	Single Model (M_O)	M_E100	M_E150	M_E225	Ensemble	Outcome
(a)	Shah-i-Zinda (12)	12 (0.78)	12 (0.84)	12 (0.81)	12 (0.72)	12 (0.90) ✓	All members agree
(b)	Ulugh Beg Madrasa (10)	10 (0.55)	10 (0.63)	7 (0.41) ✗	10 (0.58)	10 (0.72) ✓	Ensemble outvotes a dissenting member
(c)	Sherdor Madrasa (7)	10 (0.44) ✗	7 (0.52)	7 (0.57)	7 (0.49)	7 (0.72) ✓	Recovers where single model fails
(d)	Tillya-Kori Madrasa (8)	8 (0.66)	8 (0.74)	8 (0.71)	8 (0.60)	8 (0.85) ✓	All members agree
(e)	Bibi-Khanym Mosque (2)	10 (0.38) ✗	2 (0.46)	2 (0.43)	7 (0.35) ✗	2 (0.56) ✓	Narrow recovery on hardest crop
(f)	Gur-e-Amir Mausoleum (3)	3 (0.86)	3 (0.90)	3 (0.88)	3 (0.82)	3 (0.95) ✓	Distinctive dome; easy case
(g)	Imom Motrudiy Complex (5)	5 (0.64)	5 (0.72)	5 (0.70)	5 (0.61)	5 (0.82) ✓	Distinctive dome despite occlusion
(h)	Ruhobod Complex (6)	4 (0.44) ✗	6 (0.43)	4 (0.42) ✗	4 (0.40) ✗	4 (0.50) ✗	Honest failure; one correct member outvoted
